# Prevalence and Determinants of Vitamin D Deficiency in 9595 Mongolian Schoolchildren: A Cross-Sectional Study

**DOI:** 10.3390/nu13114175

**Published:** 2021-11-21

**Authors:** Jorick Bater, Sabri Bromage, Tuyatsetseg Jambal, Enkhjargal Tsendjav, Enkhsaikhan Lkhagvasuren, Yanjmaa Jutmann, Adrian R. Martineau, Davaasambuu Ganmaa

**Affiliations:** 1 Department of Nutrition, Harvard T.H. Chan School of Public Health, Boston, MA 02115, USA; sab742@mail.harvard.edu (S.B.); gdavaasa@hsph.harvard.edu (D.G.); 2 T School of Industrial Technology, Mongolian University of Science & Technology, Ulaanbaatar 14210, Mongolia; tuyatsetseg_j@must.edu.mn; 3 The Mongolian Health Initiative, Ulaanbaatar 14210, Mongolia; enkhjin2005@gmail.com; 4 Ministry of Health, Ulaanbaatar 14210, Mongolia; enkhsaikhan@mnums.edu.mn; 5 Department of Immunology, School of Biomedicine, Mongolian National University of Medical Sciences, Ulaanbaatar 14210, Mongolia; 6 Department of Math and Statistics, University of North Carolina at Charlotte, Charlootte, NC 28223, USA; yjutmaan@uncc.edu; 7 Blizard Institute, Barts and The London School of Medicine and Dentistry, Queen Mary University of London, London E1 4NS, UK; a.martineau@qmul.ac.uk; 8 Channing Division of Network Medicine, Department of Medicine, Brigham and Wome’s Hospital, Harvard Medical School, Boston, MA 02115, USA

**Keywords:** Mongolia, schoolchildren, vitamin D, determinants, serum 25(OH)D, fortification

## Abstract

Population-based data relating to vitamin D status of children in Northeast Asia are lacking. We conducted a cross-sectional study to determine the prevalence and determinants of vitamin D deficiency in 9595 schoolchildren aged 6–13 years in Ulaanbaatar (UB), the capital city of Mongolia. Risk factors for vitamin D deficiency were collected by questionnaire, and serum 25-hydroxyvitamin D (25[OH]D) concentrations were measured using an enzyme-linked fluorescent assay, standardized and categorized as deficient (25[OH]D <10 ng/mL) or not. Odds ratios for associations between independent variables and risk of vitamin D deficiency were calculated using multivariate analysis with adjustment for potential confounders. The prevalence of vitamins D deficiency was 40.6% (95% CI 39.7% to 41.6%). It was independently associated with female gender (adjusted odds ratio [aOR] for girls vs. boys 1.23, 95% CI 1.11–1.35), month of sampling (aORs for December–February vs. June–November 5.28 [4.53–6.15], March–May vs. June–November 14.85 [12.46–17.74]), lower levels of parental education (P for trend <0.001), lower frequency of egg consumption (P for trend <0.001), active tuberculosis (aOR 1.40 [1.03–1.94]), household smoking (aOR 1.13 [1.02 to1.25]), and shorter time outdoors (P for trend <0.001). We report a very high prevalence of vitamin D deficiency among Mongolian schoolchildren, which requires addressing as a public health priority.

## 1. Introduction

It is estimated that at least 1 billion individuals globally have sub-optimal serum 25-hydroxyvitamin D [25(OH)D] levels [1]. The 25(OH)D is the major circulating metabolite of vitamin D, widely acknowledged to be the most robust and reliable measure of vitamin D status [2]. Studies investigating vitamin D deficiency in Mongolia found a prevalence of 80.1% among Mongolian adults in the winter and 80% among reproductive-age women [3,4]. In particular, Mongols have low 25(OH)D levels, due in part to Mongolia’s high latitude, increasing amounts of air pollution, especially in the capital city of Ulaanbaatar, lack of sun exposure during winter and spring, and lack of access to vitamin D-rich food (e.g., fish and mushrooms) [5,6,7]. To address these issues, the Mongolian government has been considering solutions for the problem of vitamin D deficiency and ways that might promote the supply of micronutrients (like vitamin D) to the general population.

Vitamin D supplementation has been proposed as an intervention that would raise serum 25(OH)D levels. However, the most recent national nutrition survey suggests a lack of adherence to both supplements and a resistance of consumption of vitamin D-rich foods [8]. Therefore, food fortification has been widely supported recently in Mongolia, as a means of supplying vitamin D on a national scale [9]. Although fortification can be a strong tool for alleviating micronutrient deficiency, other risk factors within the Mongolian population may have major links to vitamin D deficiency in Mongolia.

The present study describes a cross-sectional analysis of vitamin D status in a large sample of Mongolian schoolchildren. These populations are of major interest because they undergo rapid growth and development. Our main purpose was to evaluate relationships that may exist between modifiable or non-modifiable risk factors and risk of vitamin D deficiency within this population, particularly household, nutritional, health, and sociodemographic determinants. Mongolia has one of the highest Tuberculosis (TB) incidence rates among Asian countries at 428 cases per 100,000 per year, out of which 10% is pediatric [10], motivating the study to assess its potential relationship with vitamin D status. We used cohort information to conduct a secondary analysis and to identify potential risk factors associated with low 25(OH)D levels. Cross-sectional studies evaluating determinants of vitamin D deficiency can inform the design of health programs by identifying risk factors that are potentially amenable to intervention. These results may enhance the effectiveness of the Mongolian government’s efforts to improve the population’s vitamin D status and help develop targeted interventions

## 2. Methods

### 2.1. Study Design and Setting

Mongolia is a land-locked country located between China and Russia with a population of 3.1 million people, of whom almost half reside in the capital city, Ulaanbaatar. We conducted a cross-sectional analysis of baseline data collected from children attending 18 public schools (located in six districts of Ulaanbaatar) who were being screened for participation in a randomized, controlled trial of vitamin D supplementation for the prevention of latent Tuberculosis infection (LTBI) [11]. As recruits to a double-blind clinical trial, participating children were randomized to receive either vitamin D supplements or placebo. This study was implemented to explore independent associations between vitamin D deficiency and increased susceptibility to TB among the Mongolian population [11]. The study was approved by Institutional Review Boards at the Mongolian Ministry of Health and the Harvard T. H. Chan School of Public Health (IRB ref. no. 14-0513) and funded by the National Institutes of Health (ClinicalTrials.gov number, NCT02276755).

### 2.2. Sample Size and Eligibility

**Figure 1:**
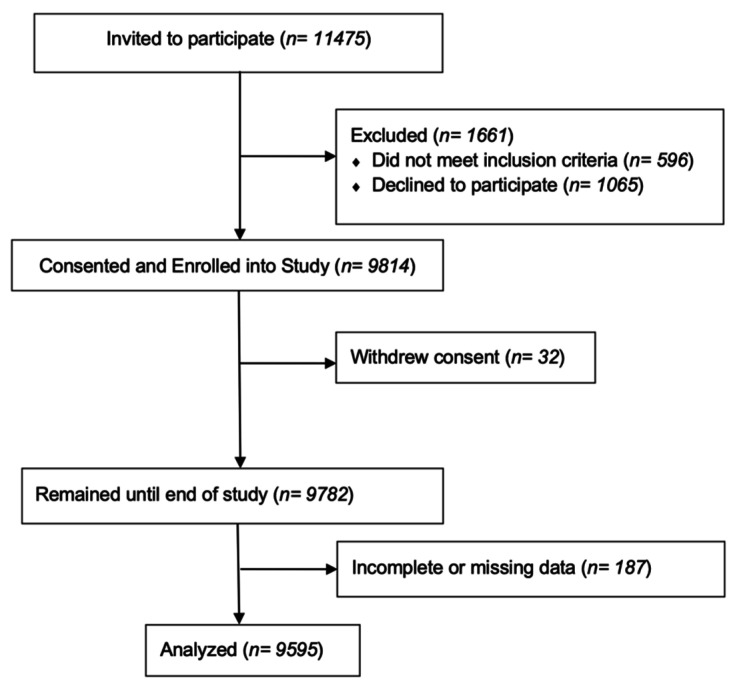
Number of individuals included and excluded at each stage of the study.

Demographic data were collected from 11,475 children in 18 public schools in Ulaanbaatar who were considered potentially eligible for the primary randomized, controlled trial. Various subjects were eliminated from further participation in the study because they did not attend informational meetings at the school or because initial consent was later retracted by parents or other legal guardians. Thus, by the end of the study only 9782 subjects remained. The subjects were children between the ages of 6 and 13 (given vitamin D supplements via school during the parent trial) at screening who attended one of the 18 schools. Inclusion criteria primarily consisted of children aged 6 to 13 years at screening and attendance at a participating school with data available, and children who had pre-existing tuberculosis or evidence of rickets were excluded. Figure 1 shows the number of children from the original sample who were included in the final sample.

### 2.3. Data Collection and Measurement

Data were collected at baseline for a parent clinical trial [11]. Household and diet information was collected at baseline via questionnaire and interview (with certain foods such as meat, fish, and eggs included as primary sources of dietary vitamin D), and parents and guardians were contacted for full details when needed. Characteristics were considered modifiable if they were behaviors or other factors that can be reasonably altered by the parent or child (e.g., district, household income, eating behaviors, physical activity, BMI-for-age, smoking levels); if not, they were considered non-modifiable (e.g., age, gender, month of sampling, TB classification). Physical measurements, vitamin D measurements, and TB tests were collected by trained trial field workers. A 5-mL blood sample was obtained from each child for QuantiFERON –TB Gold (QFT-G) testing and for measurement of serum 25(OH)D levels. Children with positive QFT-G tests were referred to the Mongolian National Center for Communicable Diseases (NCCD) for clinical and radiographic screening for tuberculosis disease. Vitamin D levels were measured in Global lab using an enzyme-linked fluorescent assay (VIDAS 25OH Vitamin D total; Biomerieux, Marcy-l’Etoile, France). The assay was accredited by the Vitamin D External Quality Assessment Scheme (DEQAS). The total Coefficient of Variation (CV) was 7.9%, mean bias was 7.7%. and the limit of quantitation (LOQ) was 8.1 ng/mL.

### 2.4. Statistical Analysis

Serum 25(OH)D levels were standardized with the use of standards provided by the Vitamin D External Quality Assessment Scheme [12] prior to conversion to a binary variable, in which vitamin D deficiency was defined as a serum 25(OH)D level less than 10 ng/mL, supported by most commercial laboratories as the standard [13]. For continuous variables (age, height, weight, waist circumference, BMI-for-age Z-score, and fat mass), means and standard deviation are reported in Table 1. Household annual income was categorized based on quartiles. BMI (body mass index) data were generated using height and weight measurements and converted to BMI-for-age Z-scores, using World Health Organization reference data via the Canadian Pediatric Endocrine Group ShinyApps platform [14]. Complete case analysis (i.e., exclusion of subjects with missing data) was performed for any individual whose record showed missing data, leaving 9595 subjects left for analysis.

The potential predictors of vitamin D deficiency were chosen based on existing literature, and variation inflation factors (a metric used to detect multicollinearity by testing how variation is inflated for a variable) were calculated to reduce the influence of highly correlated variables. Reference levels of the measured variables were chosen based on which category had the highest level of vitamin D deficiency because interpretation of odds ratio is more logical when comparing the odds to the odds of the group most vitamin D deficient. Parameter value estimates and 95% confidence limits were generated by logistic regression models. Consequently, the exponentiated value of the coefficient should be interpreted as the expected change in the odds of vitamin D deficiency in response to a one-unit increase in the level of a continuous parameter or a one-level increase in a categorical parameter, holding other parameters’ levels constant. Considering that 25(OH)D levels can greatly vary by season in Mongolia [3], a categorical variable for the month that samples were drawn was included in every model. Since summer months were not available in this dataset, months were separated into three general categories (September–November, December–February, and March–May) to capture seasonal effects. Univariate analysis was conducted for all potential predictors, and variables that yielded a *p*-value less than 0.1 were used in multivariable analysis. Those variables in the multivariable analysis that had a *p*-value less than 0.05 were considered likely determinants of vitamin D deficiency. For categorical variables, a likelihood ratio test was used to generate a global *p*-value to assess categories as a group. All analyses were done in R version 4.0 for Mac OS X Catalina, and anonymized raw data and modeling code are available on request.

## 3. Results

### 3.1. Characteristics of the Study Population

Household and demographic characteristics are detailed based on the overall population in Table 1 and among vitamin D-deficient individuals in Table 2. After removing all subjects with missing data, 9595 subjects remained to be analyzed. The overall prevalence of vitamin D deficiency (defined using the 10-ng/mL 25(OH)D threshold) in this sample was 40.6% (3900 out of 9595) (95% CI 39.7% to 41.6%). The participants’ gender distribution was virtually even, and the mean age was 9.4 years. Participants from six districts were studied, with the minority coming from an “Other” region not defined and the majority coming from the Sukhbaatar region. Most participants lived in a house or apartment without central heating or lived in ger (a traditional Mongolian felt-covered structure) and had a household income in the highest quartile of the study population. Most subjects consumed red meat every day or almost every day. In contrast, most subjects consumed eggs only 1–4 times per month and seldomly consumed any seafood and/or animal liver/intestinal organs. Most subjects did not live with any household members who smoked and did not smoke themselves. Most subjects had a BMI-for-age Z-score of between −2 and +2 but had less than 1 h of daily outdoor activity.

### 3.2. Predictors of Vitamin D Deficiency

**Figure 2:**
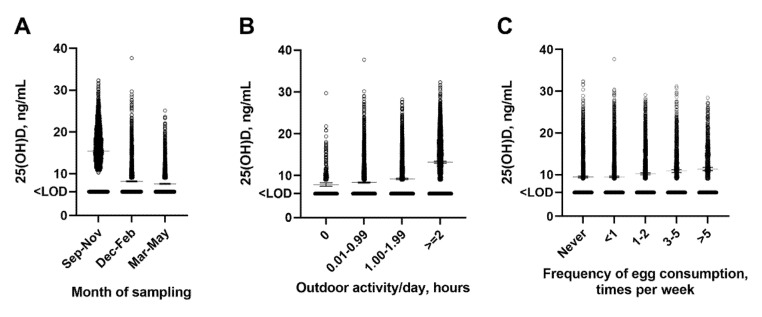
The 25(OH)D levels (ng/mL) Scatter Plots across Key Determinants (month of sampling (**A**), daily outdoor activity (**B**), and frequency of egg consumption (**C**)). Legend: Bars and whiskers of plot represent mean and 95% CI. Respectively; LOD = limit of detection.

Results of the multivariable regression analysis are summarized in Table 2. Following univariate analysis, the adjusted model included age, gender, month of sampling, district, highest level of parental education, frequency of egg consumption, TB classification, any smoking within household, and daily outdoor activity. Vitamin D deficiency was independently associated with female gender (adjusted odds ratio [aOR] for girls vs. boys 1.23, 95% CI 1.11 to 1.35), month of sampling (aOR for December–February vs. June–November 5.28, 95% CI 4.53 to 6.15, March–May vs. June–November 14.85, 95% CI 12.46 to 17.74), the districts of residence (aOR for Bayanzurkh vs. Bayangol 3.61, 95% CI 2.80 to 4.66; Chingeltei vs. Bayangol 3.46 95% CI 2.65 to 4.55, Sukhbaatar vs. Bayangol 2.78, 95% CI 2.06 to 3.77), lower levels of parental education (aOR for secondary education vs. university 1.36, 95% CI 1.21 to 1.52, primary vs. university 1.32, 95% CI 1.04 to1.69, no education vs. university 1.48, 95% CI 1.11 to 1.99), and other ORs seen from Table 2 with *p*-value below 0.05. Thus, there was a relatively even split of modifiable and non-modifiable independent risk factors that met the significance threshold. In addition, variation in 25(OH)D can be seen when investigating scatterplots with month of sampling, outdoor activity, and frequency of egg consumption on the other axis (Figure 2).

## 4. Discussion

Although predictors of vitamin D deficiency are complex, their identification is important for the development of policy that seeks to improve serum 25(OH)D levels in the Mongolian population. Using data from the parent vitamin D supplementation trial, we identified several predictors that were associated with vitamin D deficiency, including gender, months sampled, district of residency, parental education, frequency of egg consumption, TB status, any smoking in the household, and frequency of daily outdoor activity. Identification of these predictors can guide policy and program changes that may be directed towards addressing vitamin D levels in Mongolia.

The risk factors identified were independent predictors of vitamin D deficiency and consisted of an even split of modifiable and non-modifiable risk factors. District of residence in Ulaanbaatar as a risk factor is difficult to concretely explain, since there are numerous qualities of a district that may affect vitamin D status (e.g., number of markets selling vitamin D foods, traffic congestion motivating individuals to stay indoors, urban planning of outdoor space available, socioeconomic status of district). However, annual household income and type of household were not strongly associated with vitamin D deficiency, reducing the likelihood of an economic-based explanation. The months that serum was sampled had both positive and negative associations, reflecting the fluctuating nature of 25(OH)D level based on season, likely due to sunlight and food availability. Households with smokers had higher odds of vitamin D deficiency, which could be due to poorer health habits or an undefined biological relationship between secondhand smoke and 25(OH)D. This relationship has been seen before in U.S. and Korean populations and has been hypothesized to be due to nicotine-induced hypoparathyroidism that affects 25(OH)D metabolism [15,16]. A positive TB diagnosis was associated with higher odds of vitamin D deficiency, which could potentially be explained by TB interrupting healthy behavior or an undefined biological relationship (e.g., TB may potentially perturb vitamin D metabolism) [17]. Since these data are cross-sectional, we cannot exclude the possibility that vitamin D deficiency may be a consequence of TB, which should be explored in future studies. Higher frequency of egg consumption was associated with lower odds of vitamin D deficiency, which has been corroborated as a good source of vitamin D in the Asian region [18] due to a limited number of foods naturally containing vitamin D being produced in this area of the world [19]. Higher frequency of daily outdoor activity was associated with lower odds of vitamin D deficiency, which is expected given the increased exposure to sunlight. Differences in the risk of vitamin D deficiency across genders could suggest gender-specific behavioral activity or nutrition that we were unable to adjust for in the multivariable analysis. In addition, since households with parents with a university degree had a lower odds of vitamin D deficiency, it could underscore better awareness of nutrition and nutrition practices in such households.

Since these predictors reflect several underlying causes, they are difficult to address in a single policy or program. Although some modifiable predictors can be targeted via government intervention, tangible improvement necessitates a more comprehensive plan of action. In addition, micronutrient supplementation with vitamin D, although effective in raising vitamin D levels in schoolchildren [11,20,21,22], is difficult to provide for those not attending school. The Coronavirus pandemic has made accessibility to micronutrient supplements especially more difficult and costly [23]. For these reasons, food fortification has become a major focus of the government. Given the country’s large nomadic population, it is difficult to ship and store supplements over a wide and sparsely populated area [24]. Fortification of staple foods such as flour is considered a more viable option since a large portion of the population purchases commercial flours throughout the year [25,26]. A projected effectiveness study found flour to be the most practical food fortification candidate (as opposed to milk and oil), given its universal consumption and centralized production [26]. Preliminary estimates made in that report suggested that such a measure would increase the intake of vitamin D considerably. Although food fortification has been successful in addressing micronutrient deficiencies in other countries, our studies indicated that targeted interventions focused on other risk factors could complement such efforts in Mongolia.

One strength of this study is the large sample of Mongolian children, a group that has been rarely studied in the past. A wide range of household and demographic characteristics were studied, although some categories may have had too few subjects for definitive analysis. Fortunately, our rate of missing data was minimal. Another strength is that serum 25(OH)D levels took into account the month of sampling, since it has been seen that seasons have different impacts on food availability and sunlight exposure. For example, a study of pregnant women in Ulaanabatar by Uush and colleagues found that serum 25(OH)D levels varied by season [27]. This seasonal variation was also found by Bromage and colleagues among Mongolian adults, with levels in the winter being especially low [3]. Further studies should be conducted in regions outside of Ulaanbaatar for a more comprehensive look at the situation at a national level. In addition, following this same cohort could provide useful information as to how vitamin D levels change through adulthood. Current micronutrient deficiencies (including vitamin D) are ubiquitous during childhood in Mongolia and may ultimately impact growth and development and adult productivity [28]. For this reason, focusing on improving these deficiencies early through targeted interventions is of major importance for future generations.

## 5. Conclusions

Despite efforts to improve serum 25(OH)D levels in Mongolia, vitamin D deficiency remains a significant public health concern. Especially during times when infectious diseases are prevalent, like the COVID-19 pandemic, achieving and maintaining vitamin D sufficiency is of major importance. This study identified several important modifiable and non-modifiable determinants associated with vitamin D deficiency, including gender, months sampled, district of residency, parental education, frequency of egg consumption, TB status, any smoking in the household, and frequency of daily outdoor activity. The effectiveness of efforts aimed at improving 25(OH)D levels in Mongolia such as food fortification can be supplemented by targeted interventions that address determinants we identified in this study.

## Figures and Tables

**Table 1:** Characteristics of Study Population.

	Total (*N* = 9592) *n* (%)
**Age, years**	
Mean (SD)	9.41 (1.58)
Median [Min, Max]	9.24 [5.84, 14.2]
**Gender**	
Female	4764 (49.7%)
Male	4828 (50.3%)
**Month of sampling**	
Sep–Nov	2589 (27.0%)
Dec–Feb	4169 (43.5%)
March–May	2834 (29.5%)
**District**	
Bayangol	565 (5.9%)
Bayanzurkh	2319 (24.2%)
Chingeltei	1528 (15.9%)
Khan-Uul	1085 (11.3%)
Songino-Khairkhan	3430 (35.8%)
Sukhbaatar	651 (6.8%)
Other	14 (0.1%)
**Highest level of parental education ^1^**	
University	3202 (33.4%)
Technical/polytechnic/diploma	469 (4.9%)
Secondary	5104 (53.2%)
Primary	482 (5.0%)
None	335 (3.5%)
**Household income quartile (USD) ^2,3^**	
Quartile 4 (3507–7015)	3168 (33.0%)
Quartile 3 (245–3507)	2111 (22.0%)
Quartile 2 (175–245)	2337 (24.4%)
Quartile 1 (0–175)	1976 (20.6%)
**Type of accommodation**	
Centrally Heated	2219 (23.1%)
Not Centrally Heated	3731 (38.9%)
Ger With Fence	3197 (33.3%)
Ger Without Fence	445 (4.6%)
**Frequency of egg consumption**	
Every day or almost every day	841 (8.8%)
3–5 times per week	1107 (11.5%)
1–2 times per week	2250 (23.5%)
1–4 times in past month	3292 (34.3%)
None	2102 (21.9%)
**Frequency of liver/internal organs’ consumption**	
None	5002 (52.1%)
1–4 times in past month	3701 (38.6%)
1–2 times per week	653 (6.8%)
3–5 times per week	135 (1.4%)
Every day or almost every day	101 (1.1%)
**Frequency of red meat consumption**	
Every day or almost every day	9280 (96.7%)
3–5 times per week	153 (1.6%)
1–2 times per week	63 (0.7%)
1–4 times in past month	42 (0.4%)
None	54 (0.6%)
**Frequency of fish or seafood consumption**	
Every day or almost every day	78 (0.8%)
3–5 times per week	133 (1.4%)
1–2 times per week	582 (6.1%)
1–4 times in past month	2643 (27.6%)
None	6156 (64.2%)
**TB classification**	
No TB	8643 (90.1%)
Latent TB	569 (5.9%)
Active TB	270 (2.8%)
Indeterminate	110 (1.1%)
**Any smoking inside household**	
No	6084 (63.4%)
Yes	3508 (36.6%)
**Subject actively smoking**	
No	9544 (99.5%)
Yes	48 (0.5%)
**BMI-for-age Z-score**	
≤2.00 (underweight)	114 (1.2%)
−2.00 to 2.00 (normal range)	8938 (93.2%)
2.01 to 3.00 (overweight)	428 (4.5%)
>3.00 (obese)	97 (1.0%)
**Frequency of Outdoor Activity**	
Greater than 2 h	2628 (27.4%)
1–2 h	2934 (30.6%)
Less than 1 h	3725 (38.8%)
None	305 (3.2%)

^1^ Refers to highest level of education for either parent. ^2^ Abbreviations: USD = US Dollar, SD = standard deviation, BMI = Body Mass Index, TB = tuberculosis. ^3^ This calculation was conducted using the current exchange rate between Tugrik and USD at time of writing (i.e., 1 USD = 2848 Tugrik).

**Table 2:** Determinants of vitamin D status.

		Univariate Analysis	Multivariable Analysis		
	Proportion with 25(OH)D <10 ng/mL (%)	Crude OR ^1^ (95% CI)	Adjusted OR (95% CI) ^2^	*p*	*P* for Trend
**Age (years) ^3^**		1.07 (1.04, 1.09)	1.09 (1.05,1.13)	<0.001	
6.00–6.99	73/254 (28.7%)	N/A	N/A		
7.00–7.99	1099/1946 (56.5%)	N/A	N/A		
8.00–8.99	1325/2183 (60.7%)	N/A	N/A		
9.00–9.99	987/1658 (59.5%)	N/A	N/A		
10.00–10.99	1045/1625 (64.3%)	N/A	N/A		
11.00–11.99	900/1046 (86.0%)	N/A	N/A		
12.00–12.99	245/488 (50.2%)	N/A	N/A		
13.00–13.99	18/32 (56.3%)	N/A	N/A		
**Gender**					
Male	2792/4828 (57.8%)	Ref	Ref		
Female	2900/4764 (60.9%)	1.13 (1.05, 1.23)	1.23 (1.11,1.35)	<0.001	
**Month of sampling**					
Sep–Nov	452/2589 (17.5%)	Ref	Ref		
Dec–Feb	3030/4169 (72.7%)	12.58 (11.14, 14.23)	5.28 (4.53, 6.15)	<0.001	
March–May	2210/2834 (78.0%)	16.74 (14.65, 19.18)	14.85 (12.46, 17.74)	<0.001	
**District**					
Bayangol	110/565 (19.5%)	Ref	Ref		
Bayanzurkh	1707/2319 (73.6%)	11.54 (9.22, 14.55)	3.61 (2.80, 4.66)	<0.001	
Chingeltei	1171/1528 (76.6%)	13.57 (10.71, 17.31)	3.46 (2.65, 4.55)	<0.001	
Khan-Uul	266/1085 (24.5%)	1.34 (1.05, 1.73)	0.91 (0.7, 1.2)	0.50	
Songino-Khairkhan	1963/3430 (57.2%)	5.53 (4.46, 6.92)	1.04 (0.8, 1.35)	0.78	
Sukhbaatar	469/651 (72.0%)	10.66 (8.178, 14.01)	2.78 (2.06, 3.77)	<0.001	
Other	6/14 (42.9%)	3.10 (1.00, 9.10)	0.93(0.27, 3.2)	0.91	
**Highest level of parental education**					
University	1564/3202 (48.8%)	Ref	Ref		
Technical/polytechnic/diploma	276/469 (58.8%)	1.50 (1.23, 1.82)	1.24 (0.97, 1.58)	0.09	<0.001
Secondary	3288/5104 (64.4%)	1.90 (1.73, 2.07)	1.36 (1.21, 1.52)	<0.001	
Primary	328/482 (68.0%)	2.23 (1.82, 2.74)	1.32 (1.04, 1.69)	0.03	
None	236/335 (70.4%)	2.49 (1.96, 3.20)	1.48 (1.11, 1.99)	0.01	
**Household income quartile**					
Quartile 4	1577/3168 (49.8%)	Ref	Ref		
Quartile 3	1275/2111 (60.4%)	1.54 (1.38, 1.72)	N/A	N/A	N/A
Quartile 2	1502/2337 (64.3%)	1.81 (1.63, 2.03)	N/A	N/A	
Quartile 1	1338/1976 (67.7%)	2.12 (1.88 2. 40)	N/A	N/A	
**Type of accommodation**					
Centrally Heated	963/2219 (43.4%)	Ref	Ref		
Not Centrally Heated	2357/3731 (63.2%)	2.49 (2.02, 3.09)	N/A	N/A	N/A
Ger With Fence	2080/3197 (65.1%)	2.42 (2.17, 2.71)	N/A	N/A	
Ger Without Fence	292/445 (65.6%)	2.24 (2.01, 2.49)	N/A	N/A	
**Frequency of egg consumption**					
Every day or almost every day	378/841 (44.9%)	Ref	Ref		
3–5 times per week	569/1107 (51.4%)	1.30 (1.08, 1.55)	1.40 (1.13, 1.73)	<0.001	<0.001
1–2 times per week	1283/2250 (57.0%)	1.63 (1.39, 1.91)	1.54 (1.27, 1.86)	<0.001	
1–4 times in past month	2101/3292 (63.8%)	2.16 (1.85, 2.52)	1.92 (1.60, 2.31)	<0.001	
None	1361/2102 (64.7%)	2.25 (1.91, 2.65)	1.98 (1.62, 2.41)	<0.001	
**Frequency of liver/internal organs consumption**					
None	2945/5002 (58.9%)	Ref	Ref		
1–4 times in past month	2180/3701 (58.9%)	1.00 (−0.92, 1.09)	N/A	N/A	N/A
1–2 times per week	408/653 (62.5%)	1.16−0.98, 1.38)	N/A	N/A	
3–5 times per week	86/135 (63.7%)	1.23 (−0.86, 1.76)	N/A	N/A	
Every day or almost every day	73/101 (72.3%)	1.82 (1.19, 2.87)	N/A	N/A	
**Frequency of red meat consumption**					
Every day or almost every day	5511/9280 (59.4%)	Ref	Ref		
3–5 times per week	89/153 (58.2%)	0.95 (0.69, 1.32)	N/A	N/A	N/A
1–2 times per week	34/63 (54.0%)	0.80 (0.49, 1.33)	N/A	N/A	
1–4 times in past month	26/42 (61.9%)	1.11 (0.60, 2.12)	N/A	N/A	
None	32/54 (59.3%)	0.99 (0.58, 1.74)	N/A	N/A	
**Frequency of fish or seafood consumption**					
Every day or almost every day	27/78 (34.6%)	Ref	Ref		
3–5 times per week	58/133 (43.6%)	1.46 (0.82, 2.63)	N/A	N/A	N/A
1–2 times per week	299/582 (51.4%)	2.00 (1.23, 3.31)	N/A	N/A	
1–4 times in past month	1482/2643 (56.1%)	2.41 (1.52, 3.92)	N/A	N/A	
None	3826/6156 (62.2%)	3.10 (1.96, 5.03)	N/A	N/A	
**TB classification**					
No TB	5074/8643 (58.7%)	Ref	Ref		
Latent TB	364/569 (64.0%)	1.25 (1.05, 1.49)	0.99 (0.80, 1.23)	0.93	
Active TB	194/270 (71.9%)	1.80 (1.38, 2.36)	1.40 (1.03, 1.94)	0.04	
Indeterminate	60/110 (54.5%)	0.84 (0.58, 1.23)	0.75 (0.48, 1.2)	0.22	
**Any smoking inside household**					
No	3454/6084 (56.8%)	Ref	Ref		
Yes	2238/3508 (63.8%)	1.34 (1.23, 1.46)	1.13 (1.02, 1.25)	0.02	
**Subject actively smoking**					
No	5667/9544 (59.4%)	Ref	Ref		
Yes	25/48 (52.1%)	0.74 (0.42, 1.31)	N/A	N/A	
**BMI-for-age Z-score**					
<−2.00	61/114 (53.5%)	Ref	Ref		
−2.00 to 1.99	5332/8938 (59.7%)	1.28 (0.88, 1.86)	N/A	N/A	N/A
2.00 to 3.00	239/428 (55.8%)	1.10 (0.72, 1.66)	N/A	N/A	
>3.00	51/97 (52.6%)	0.96 (0.56, 1.66)	N/A	N/A	
**Frequency of Daily Outdoor Activity**					
Greater than 2 h	932/2628 (35.5%)	Ref	Ref		
1–2 h	1872/2934 (63.8%)	3.21 (2.87, 3.58)	1.50 (1.30, 1.72)	<0.001	<0.001
Less than 1 h	2659/3725 (71.4%)	4.54 (4.08, 5.05)	1.68 (1.47, 1.93)	<0.001	
None	229/305 (75.1%)	5.48 (4.20, 7.23)	1.72 (1.27, 2.34)	<0.001	

^1^ Abbreviations: US Dollar, SD = standard deviation, BMI = Body Mass Index, TB = tuberculosis, OR = Odds Ratio, Ref = reference, CI = confidence interval, N/A = not available, P = probability, ng = nanograms, mL = milliliters. ^2^ Variables were adjusted for by all other variables with *p*-value < 0.1 from univariate analysis. ^3^ Age as a whole is included in the model as a continuous variable but is listed in the table based on age band.

## Data Availability

The data presented in this study are available on request from the corresponding author. The data are not publicly available due to privacy restrictions.
